# Predicting age groups of Twitter users based on language and metadata features

**DOI:** 10.1371/journal.pone.0183537

**Published:** 2017-08-29

**Authors:** Antonio A. Morgan-Lopez, Annice E. Kim, Robert F. Chew, Paul Ruddle

**Affiliations:** 1 Behavioral Health and Criminal Justice Research Division, RTI International, Research Triangle Park, North Carolina, United States of America; 2 Center for Health Policy Science & Tobacco Research, RTI International, Berkeley, California, United States of America; 3 Center for Data Science, RTI International, Research Triangle Park, North Carolina, United States of America; Rutgers The State University of New Jersey, UNITED STATES

## Abstract

Health organizations are increasingly using social media, such as Twitter, to disseminate health messages to target audiences. Determining the extent to which the target audience (e.g., age groups) was reached is critical to evaluating the impact of social media education campaigns. The main objective of this study was to examine the separate and joint predictive validity of linguistic and metadata features in predicting the age of Twitter users. We created a labeled dataset of Twitter users across different age groups (youth, young adults, adults) by collecting publicly available birthday announcement tweets using the Twitter Search application programming interface. We manually reviewed results and, for each age-labeled handle, collected the 200 most recent publicly available tweets and user handles’ metadata. The labeled data were split into training and test datasets. We created separate models to examine the predictive validity of language features only, metadata features only, language and metadata features, and words/phrases from another age-validated dataset. We estimated accuracy, precision, recall, and F1 metrics for each model. An L1-regularized logistic regression model was conducted for each age group, and predicted probabilities between the training and test sets were compared for each age group. Cohen’s d effect sizes were calculated to examine the relative importance of significant features. Models containing both Tweet language features and metadata features performed the best (74% precision, 74% recall, 74% F1) while the model containing only Twitter metadata features were least accurate (58% precision, 60% recall, and 57% F1 score). Top predictive features included use of terms such as “school” for youth and “college” for young adults. Overall, it was more challenging to predict older adults accurately. These results suggest that examining linguistic and Twitter metadata features to predict youth and young adult Twitter users may be helpful for informing public health surveillance and evaluation research.

## Introduction

Public health organizations are increasingly using social media to disseminate messages about health to wide audiences. Campaigns targeting youth and young adults actively use social media because it is an influential source of information in the lives of youth and young adults. Determining the extent to which the target audience was reached is critical to evaluating the impact of public health social media campaigns. To do so, agencies rely on readily available analytic tools from social media platforms (e.g., Facebook Insights, Twitter Analytics) and third-party companies (e.g., Demographics Pro) that summarize audience demographic profiles. However, these tools have several limitations. First, the demographic information is not comprehensive across social media platforms and may be reported in categories that do not map to the target audience. For example, at the time this study was conducted, Twitter Analytics provided information about followers’ gender and interests but not age. Second, these analytic tools only provide demographic information about social media users who are actively following specific social media accounts (e.g., campaign Twitter handles and Facebook groups) and not about users who may be actively discussing the campaign but not following these accounts. This limits researchers’ ability to measure the true reach of their campaign efforts. Third, because these tools are proprietary, the methodological approach used to infer age or other demographic characteristics of social media users is unknown. Increasingly, researchers in computer science and other disciplines are developing methods to predict the age and demographic characteristics of social media users based on publicly available information from users’ profiles and post content (e.g., [1–3]).

One way to predict age and other demographic information uses differences in linguistics to infer age groupings [4–9]. Vocabulary, writing style, and speech patterns evolve over time as individuals learn and develop [10], and linguists have marked specific linguistic milestones that distinguish language use during childhood, adolescence, and adulthood [11]. Empirical studies that have linked variation in language use and age include the analysis of phone conversations, blog postings, online reviews, Facebook posts, and Twitter tweets [12–17].

The most comprehensive studies linking language use and demographics in social media data emerge from the work of Schwartz and colleagues [4] as part of the World Well-Being Project (WWBP). Within the WWBP, investigators have used an open vocabulary analysis framework, whereby they link a series of individual words, phrases, and topics that emerge from open text context from Facebook posts and comments and correlate the groupings of words with known features, such as age, gender, and personality, from a survey of ~75,000 participants. In this work, WWBP investigators have shown clear distinctions across age groupings (e.g., ages 13–18, 19–22, 23–29, 30–65) in the use of specific words and terms that reflect (a) the greater use of emoticons and slang among younger groups and (b) the developmental progression of individuals at different life stages (e.g., school, college, career, marriage, children, family).

However, with the exception of Al Zamal et al. [15], these studies had labeled data with known ages from external information (e.g., surveys, user profiles) [5, 12, 18]. Such labeled demographic data in general, and age data in particular, are not systematically collected by Twitter when users set up new accounts. Furthermore, the comprehensive linguistic database from the WWBP study is unique to Facebook, and researchers [4] have cautioned against generalizing Facebook linguistic tendencies to Twitter in computational linguistic analysis because the 140 character limit of tweets may constrain language use in such a way that it does not reflect how they use language in an unrestricted context.

A complementary approach to generating age data when labeled data are unavailable in Twitter is referred to as age annotation [13, 15]. Nguyen et al. [13] and Al Zamal et al. [15] searched the Twitter application programming interface (API) to identify Twitter accounts that had tweets about birthdays that also mentioned the age of the person: either individuals who tweeted about their own birthdays (e.g., “Happy XX birthday to me!”) or individuals who sent birthday wishes to others (e.g., “Wishing @xxxxxx a happy XX birthday”). Nguyen et al. also used age from adjoining LinkedIn profiles and estimated age for youth who tweeted about a particular grade level in school. However, approaches that combine the use of age-annotated data are still in their infancy, and these methods have not been widely applied to predict age of Twitter users. For example, Nguyen et al. [13] used the Linguistic Inquiry and Word Count (LIWC) approach (for Dutch samples, Zijlstra et al. [19]), but the open vocabulary framework used by Schwartz et al. [4] yields superior predictive power to the LIWC approach in analyzing U.S. Facebook samples.

*Predicting demographics from metadata*. Other investigators have used metadata, such as characteristics of the Twitter profile (that are independent of tweet content), to predict demographic information. For example, Rao et al. [18] examined profile statistics, such as the number of followers, the number of profiles the person followed, and the ratio of followers-to-following, but found “no exploitable differences” in the distributions of the demographic characteristics examined (e.g., gender, age, political affiliation). However, in this example, the coarse categorization of two age groupings using age 30 as the threshold between two groups may have been too crude to capture variation in age. Alowibdi et al. [20] assessed whether profile features, such as background color, text color, and border color, were predictive of demographic characteristics, although the primary focus was on predicting gender. Sloan et al. [21] examined information in individuals’ Twitter profile descriptions to identify their professions and linked those professions to the UK’s Standard Occupational Classification 2010 demographic breakdowns to predict the users’ age group. However, they reported a 42.2% error rate in predicting age with this approach. In fact, Sloan et al. [21] and Rao et al. [18] suggest that combining the predictive power of metadata with linguistics may be more powerful than either approach alone; researchers could potentially increase the application and utility of the age prediction tools by also examining language use (e.g., word use, emoticons, URLs) to build predictive models similar to those developed by Schwartz et al. [4] but tailored to Twitter data. A recent study compared the predictive power of profile features vs. linguistic features in predicting Twitter users’ income [1], but we are not aware of similar studies for predicting age.

*The present study*. The objective of the present study was to assess the separate and joint predictive validity of linguistic and metadata approaches to age prediction, given calls to examine the joint predictive power of both approaches. We couple the two predictive approaches with the age annotation and labeling approach of Al Zamal et al. [15] and Nguyen et al. [13] to develop predictive approaches to age groupings that are reflective of an interest in distinguishing between youth (ages 13–17), “emerging” young adults (ages 18–24) [22], and adults aged 25 or older. We anticipated that the combination of approaches would increase the age prediction validity in Twitter data at a rate that is significantly higher than either approach alone. We employ a holdout-validation approach to our data and analysis, by which we divide our sample into (a) a training dataset, where we estimate model parameters in the prediction of age categories conditional on linguistics and metadata; and (b) a test dataset, where the linguistics and metadata parameters estimated in the training dataset were applied to the test dataset, and the predicted age categories were compared with the actual age categories. Our study addresses several limitations in the literature on age prediction in Twitter data. First, it is not yet clear whether linguistic differences across age in social media platforms, that have been examined primarily in Facebook, would generalize to Twitter given the differences in factors such as post sizes/character limits. Further, we examine the extent to which linguistic features can be added to the prediction of age groupings to reduce the noted error rates in using metadata alone.

## Methods

### Data collection

Birthday announcement tweets were collected from the Twitter Search API (https://api.twitter.com/1.1/search/tweets.json) using the search parameters “Happy nth Birthday.” Al Zamal et al. [15] employed a similar approach, using “Happy nth Birthday to me” to capture self-reported announcements; our generalized variant captures both self-reported birthday tweets and congratulatory tweets from other users, reaching a more diverse pool of Twitter users. Birthday tweets for ages 13 to 50 were collected on August 22, 2014, September 29, 2014, April 2, 2015, and June 21, 2015. Using multiple dates allowed us to collect a wider range of birthdays and increase the size of our dataset, but the absence of a common cross-section complicated the creation of some time-dependent variables, such as the account age or number of lifetime tweets. To help correct for this, we used user metadata and language features from the latest tweets to get the most recent values at a comparable point in time.

Each birthday tweet was manually reviewed to determine whether a user could be identified from the birthday message, to determine whether the declared age seemed reasonable (rather than a joke exaggerating the age of the user for comedic effect), and to exclude “celebrity” users whose content feed may be curated for promotional and endorsement reasons. Table 1 shows the number of unique users identified after manual review and collection of additional tweets. The most users were identified in the young adult 18 to 24 age category (1,634), followed by the youth 13–17 age group (1,036), and adults 25 or older (514). Up to the latest 200 tweets were then collected for each age-labeled handle using the Twitter REST API (https://api.twitter.com/1.1/statuses/user_timeline.json) approximately 2 weeks after initial birthday tweet collection. Although the Twitter API allows collection of up to 3200 most recent tweets, prior studies have shown that examining more than 100 to 200 posts per user provides minimal gain in model performance when predicting user demographics [8, 23].

**Table 1:** Number of unique Twitter users identified from birthday tweets by age group.

Age Group	N
Youth: 13–17	1,036
Young adults: 18–24	1,634
Adults: 25 or older	514

### Data preparation

To assess the separate and joint predictive validity of linguistic and metadata approaches to age classification, we created models using four different variable sets: (1) language features only, (2) metadata features only, (3) language and metadata features, and (4) WWBP words and phrases. A more detailed list and descriptions of features for each model can be found in S1 Table. A public dataset with derived features from our sample is available on Figshare [24].

*Language features only*. To determine how important language features are in classifying users into age categories, we created a set of variables that only require a user’s tweet text. For a given user, the tweet text for up to the last 200 tweets were pooled together and converted into a bag-of-words vector space model. We used Carnegie Mellon Ark lab’s Twonkenizer [25] to tokenize the tweets and removed common stop words (e.g., the, an) and words used in the initial search (e.g., “Birthday,” “16th”). One limitation of bag-of-words models is that they calculate term frequencies without context of neighboring words, suppressing information needed to identify multi-word phrases and homonyms. To incorporate additional context into our model, we created bigram and trigram variables that combine adjacent terms. There were a total of 7717 uni-grams, 4098 bi-grams, and 289 tri-grams that were included in the feature set. If an n-gram was used by less than 1% of users or more than 99%, it was not considered for the feature set. Other linguistic variables were considered based on popular Internet conventions, such as use of excessive capitalization or punctuation (e.g., “WHAT!?!?!”), alphabetical lengthening (e.g., “that was sickkkkk”), use of emojis, and acronyms with Internet origins (e.g., “lol,” “omg”). Finally, we included linguistic features that may be indicative of professional parlance and customs, such as the count of swear words, dictionary words, or words > 6 letters [26]. In total, we constructed 38,152 language features.

*Metadata features only*. Separate from the actual tweet content is user metadata (i.e., variables that can be developed from a user’s profile and usage patterns). Features like the number of followers, number of friends, and tweeting frequency provide us information about the level of engagement and user habits, which may be indicative of larger generational trends in adoption of social media platforms or social network norms. In total, we constructed 48 metadata features.

*Language and metadata features*. To determine how useful the combination of linguistic features and metadata are in classifying users into age categories, we created a variable set containing all the language and metadata features. This variable set provides context into how correlated and intertwined the two concepts are and provides researchers with information on the marginal benefit of gathering, preparing, and analyzing additional variables for a production age classification model.

*WWBP words and phrases*. In addition to creating models based on learned features from the current data set, we incorporated lexica from the literature that have been shown to be effective in capturing life stage differences. To provide a baseline to our work, we created a variable set using the WWBP age indicative lexicon [4]. This set additionally allowed us to assess how the open vocabulary analysis framework generalizes between social media platforms (in this case, Facebook to Twitter). For four age bins (13–18, 19–22, 23–29, 30 or older), the WWBP publishes the top 100 most positively and most negatively correlated words and phrases; words and phrases from these lists were included in a WWBP variable set, as well as grouped variables containing frequency counts of the number of top 100 terms contained in the tweet text for each age group (both positive and negative). These grouped variables were developed to include terms that individually may have low counts for any given user, but are unique to an age group. In total, we examined 336 WWBP features. We chose to use only the top terms instead of the entire WWBP lexicon because we had substantially smaller samples (hundreds) for each age group compared to the tens of thousands of people in the WWBP sample. If we used the entire WWBP lexicon, we would introduce a dimensionality problem where we would have more predictors than cases in the sample. Our decision to use the top most predictive terms reflects what might be done with smaller, specifically targeted samples, thereby making our approach more applicable to other public health campaign research.

### Data structure and statistical models

Hyperparameter tuning was performed on the models to explore the feature space and experiment with different modeling assumptions. L1, L2, and elastic net regularization was performed on the linear models and feature importance scores were considered with tree based models to help prevent overfitting. Each feature was scaled to a minimum of zero and a maximum of one. A grid search on the feature importance and regularization parameters was conducted to determine cutoffs that would perform well on the test set metrics. The labeled data set was split into two datasets; 80% of the cases were used as a training dataset for parameter estimation, and the remaining 20% was used as a test sample to generate the final model evaluation metrics. The 80/20 split is widely used in the literature (e.g., [27]). A 10-fold cross validation was performed on the training set, and the validation folds and test sets were stratified by the proportions of the target variables in the complete data set.

To model age, we tested six different classifiers (logistic regression, support vector machines, random forests, adaBoost, and extra trees) and included a dummy classifier to assess baseline performance. F1 scores were highest for the logistic regression classifier with L1 regularization (73.9%), showing a large gain when compared the dummy classifier (38%), a naïve model that makes predictions based solely on the target variable’s class distribution (Tables A-B in S2 Table). A “one-vs.-rest” strategy was employed for multinomial classification [28], generating logistic regression models for each age group to allow for comparison of precision and recall metrics and important features. To evaluate the test set, predicted probabilities between the models for each age group were compared, and each individual was assigned the age group for which it had the highest predicted probability.

## Results

*Overall model precision and recall*. Overall, the model with both Tweet language use features and metadata features performed the best (74% precision, 74% recall, F1 score 74%), with the model containing only Tweet language features also performed strongly (72% precision, 72% recall, F1 score 72%) (Table 2). The model containing only WWBP words saw a drop in performance (68% precision, 67% recall, 67% F1 score) comparably, while the model containing only Twitter metadata features had the lowest precision (58%), recall (60%), and F1 score (57%). This trend was consistent in models across all three age groups. Generally, the 18 to 24 age group had the best precision scores (61% to 80%) and the 25 or older age group had the worst (47%-63%). Recall scores were lowest in the 25 or older age category; in particular, the metadata-only model was not sensitive at identifying the older age group with only a 17% recall score.

**Table 2:** Precision and recall results from validation of multiple age classification models.

Age Group	Tweet Language Use Only			Twitter Handle Metadata Only			Tweet Language Use and Twitter Handle Metadata			WWBP Words		
	Precision	Recall	F1	Precision	Recall	F1	Precision	Recall	F1	Precision	Recall	F1
13–17	69%	71%	70%	59%	51%	55%	71%	75%	73%	62%	72%	67%
18–24	78%	74%	76%	61%	78%	68%	80%	73%	76%	77%	65%	71%
25 or older	60%	65%	65%	47%	17%	25%	63%	73%	67%	52%	59%	55%
**Overall**	**72%**	**72%**	**72%**	**58%**	**60%**	**57%**	**74%**	**74%**	**74%**	**68%**	**67%**	**67%**

WWBP = World Well Being Project [4].

*Misclassification*. The confusion matrix (Table 3) summarizes the extent of misclassification in the combined Tweet language use and metadata model on the test set. The model correctly identified 155 cases in the 13 to 17 age category, 239 cases in the 18 to 24 age category, and 74 cases in the 25 or older age category, resulting in an overall model accuracy of 73.7%. Comparing ground truth labels to the predictions, Twitter users under 18 were most often misclassified as being 18 to 24 (42 cases). Likewise, Twitter users aged 18 to 24 were most often misclassified as youth 13 to 17 (53 cases). Twitter users aged 25 or older were most likely misclassified as young adults aged 18 to 24.

**Table 3:** Confusion matrix.

		**Predicted**		
		**13 to 17**	**18 to 24**	**25 or older**
**Actual**	**13 to 17**	155	42	9
	**18 to 24**	53	239	35
	**25 or older**	11	17	74

### Top linguistic and metadata features

Table 4 summarizes top linguistic and metadata features that were most predictive in classifying the three age groups in the best performing model (i.e., tweet language use and Twitter handle metadata model). Cohen’s d effect sizes were calculated for relevant metadata and linguistic features by first converting corresponding Chi-square value into correlation coefficient (r) per the formula $\sqrt{\frac{X^{2}}{N}}$. This value was then converted into a Cohen’s d effect size per the formula $\frac{2r}{1-r^{2}}$ [29]. Top features that were predictive of youth included lower “age” of Twitter account (i.e., how long the account had been open) (Cohen’s d = 0.336), less use of the word “college” (Cohen’s d = 0.236), less use of WWBP Facebook words that were positively correlated with 23 to 29 age group (Cohen’s d = 0.222), and more use of the word “school (Cohen’s d = 0.210). Top features that were predictive of young adults included less use of WWBP Facebook words that were negatively associated with the 19 to 22 age group (Cohen’s d = 0.331), more use of the word “college” (Cohen’s d = 0.232), more use of the term “18” (Cohen’s d = 0.210), and more use of the term “21” (Cohen’s d = 0.209). Other top features that were positively associated with young adults include greater use of the words “drunkard” and “semester.” Top features in predicting adults included less use of the word “school” (Cohen’s d = 0.194), older age of Twitter account (Cohen’s d = 0.193), greater use of “via” stems, greater use of URLs in tweets, and less use of smiley emoji.

**Table 4:** Top predictive features for each age group in tweet language use and Twitter handle metadata models.

Predictive Features	Youth (Aged 13 to 17)		Young Adults (Aged 18 to 24)		Adults (Aged 25 or Older)	
*Metadata Features*	Cohen’s d	Direction of Association	Cohen’s d	Direction of Association	Cohen’s d	Direction of Association
Age of Twitter Account	0.336	−			0.193	+
***Linguistic Features***						
Count of the term “school”	0.210	**+**			0.194	−
Count of WWBP words positively correlated with 23–29 age category, in tweet	0.222	−				
Count of the stems of “ili” (e.g. “I like”)	0.186	−				
Count of the term “college”	0.236	−	0.232	**+**		
Percent of WWBP words negatively correlated with 19–22 age category, in tweet^a^	0.171	**+**	0.331	**-**		
Count of stems of 18^b^			0.210	**+**		
Count of stems of 21			0.209	**+**		
Count of the term “drunkard”			0.194	**+**		
Count of the term “semester”			0.179	**+**		
Count of kissyheart emoji			0.162	**+**		
Count of smiley emoji					0.170	-
Count of stems of “via”					0.172	+
Mean absolute deviation of count of URLs in tweet^a^					0.174	+

^a^ To capture the distributional properties of a user’s tweeting behavior, we created tweet-level features and then calculated descriptive statistics of those features across a user’s tweets. For example, for the “Average Percent Characters in Tweet that are Emoji” feature, we calculated the percentage of characters that are emoji for each tweet and then took the average across all the user’s collected tweets.

^b^ To group common categorizes of words together, terms were stemmed, a process of reducing words to their base form. For example, a stemming algorithm would reduce the words “hunting,” “hunter,” “hunts,” and “hunters” to the stem “hunt.”

## Discussion

In summary, we find that examining tweet linguistic features and Twitter handle metadata features combined is more useful in predicting age of Twitter users compared to Twitter metadata or linguistic features alone. The performance for our best model (74% precision, 74% recall, and 74% F1 score) was comparable with other three-class models (e.g., 75% accuracy in predicting three classes of socioeconomic status [3]). Additionally our results were also within range of two-class models predicting age from Twitter data. For example, Rao et al. [18] achieved accuracy of 0.74 in predicting age groups ≤30 vs. > 30. Although other studies such as Al Zamal et al. [15] achieved higher accuracy of 0.80, they examined narrower age groups: 18–23 vs. 23–25. In general, it is challenging to compare model performance across studies because of differences in age groups examined and sampling and annotation methods used. Our prediction accuracies were particularly strong for youth (13 to 17 years) and young adults (18 to 24 years). It was more challenging to predict older adults accurately; our misclassification rate was nearly 50% for individuals aged 25 to 50. The poorer performance is likely due to having fewer labeled cases for this older age group. In general, we know that older adults are less likely to be on Twitter than their younger counterparts (36% of 18- to 29-year-olds vs. 22% of 30- to 49-year-olds [30]) and probably less likely to post about their birthdays. Future studies with more balanced classes would allow us to better predict the older adult age group. However, even if we had sufficient labeled cases, we hypothesize that this older age group would be difficult to classify because the wide age range of 25 to 50 encompasses large variation in life stages, which would be reflected with substantial variability in Twitter language use and profile characteristics. Breaking this older age group into smaller age increments (e.g., 5 years or 10 years) could improve model performance. However, for the purposes of this study, we grouped anyone over age 25 into the broad “older adult” category because we were primarily interested in distinguishing youth (13–17) and young adults (18–24) given that these age groups are common targets for public health education campaigns and surveillance of risky health behaviors (e.g., alcohol, tobacco, drug use, unprotected sex). Adolescence (< 18 years) and emerging adulthood (18 to 24 years) are regarded as distinct developmental periods of change and identity exploration along with high rates of risky behaviors [22]. Since social media use is prevalent among youth and young adults [30], being able to predict these age groups online enables public health programs to better monitor emerging health issues (e.g., [31, 32]), assess whether high-risk groups are being exposed to marketing and misinformation online (e.g., [33]), and target their education campaigns (e.g., https://twitter.com/abvethinfluence/, https://twitter.com/knowtherealcost/, https://twitter.com/talkhiv) more effectively.

The present study makes a unique contribution to the literature in that we explicitly compared the predictive utility of metadata and linguistic features independently vs. combined in predicting age of Twitter users. Additionally, studies to date have not examined the accuracy between predicted and actual ages. The work from the WWBP group (e.g., [4]) has focused on assessing models through individual parameters for words across age groups but does not provide information on predictive accuracy at the person level; this approach also requires the use of thousands of words to generate predicted ages and would not be ideal if the interest was in parsimony and evaluating accuracy at the person level. Other approaches that have focused on predictive accuracy of age from metadata at the person level have found low accuracy (~42% age prediction error; Sloan et al. [21]) and have suggested that a combined linguistics/metadata approach could prove fruitful [18, 21]. Our combined linguistics/metadata approach yielded an overall age misclassification rate of 30%. This improvement in prediction is all the more striking because, unlike other studies in this area, we used a holdout validation strategy, which is more conservative than single sample approaches where parameters are estimated in one sample but are not applied to a second independent sample.

Several limitations of the study and the sample need to be noted. First, individuals who tweet about their birthdays may constitute a specific subpopulation that reflects a selection bias compared with individuals who do not tweet about their birthdays. However, all classification studies using social media data are to some extent biased since there is no comprehensive frame of all users to sample from. That said, the similarity in language use in our sample compared with other studies, such as WWBP, coupled with our relatively low misclassification rates, suggest that there may be common language patterns for youth and young adults across social media platforms.

Second, studies of this nature may need continual updating. Cohort effects in language usage (e.g., slang terms, emoji usage) may vary over time. In fact, shorthand terms like “LOL” are being used more by adults than kids, and kids are eschewing the use of abbreviations for emojis [34]. Furthermore, seasonality in topics and events relevant to different age groups may need further examination (e.g., summer school, graduation). In addition to linguistic features, metadata features may also be unstable over time as they reflect the length of the time a Twitter account was open.

Third, we identified a relatively smaller sample of older age groups (aged 25 or older), which likely explains the poorer performance in predicting this age group. One possible explanation for the smaller sample may be that older adults are less likely to be on Twitter. In 2016, 36% of adults aged 18 to 29 used Twitter, compared with 22% of adults aged 30 to 49 [30]. Additionally, older adults are probably less likely to announce birthdays publicly than their younger counterparts. In our data labeling, the number of birthday announcement tweets declined dramatically for older ages. Future studies that oversample older Twitter users and use complementary labeled age data (e.g., via survey) could produce larger samples of older ages and improve the classification accuracy for older Twitter users. Twitter’s recent change enabling users to post their birthday on their profile description could also be used as a data source [35]; however, similar biases are likely in terms of who chooses to report birthdays publicly on Twitter. Another strategy might be to use the lexica from other social media platforms that older adults more commonly use such as Facebook [30] to predict this age group on Twitter, assuming that older adults’ linguistic patterns are similar across social media platforms.

Fourth, we did not examine topic distributions as features in our model. Recent studies have shown that topic clusters are important features in predicting demographic characteristics (e.g., [1, 3]). For example, in predicting income of Twitter users, Preotiuc-Pietro and colleagues [1] achieved best results using topic clusters (correlation 0.633) compared with other types of features such as profile characteristics (correlation 0.372). A future expansion of our work would be to examine whether topic features are predictive of youth and young adult age groups on Twitter.

In conclusion, we were able to utilize Twitter linguistic patterns and metadata to predict youth and young adult age groups with relatively high accuracy. Our results suggest that models performed best with both linguistic and metadata features, and that there is some similarity in how youth and young adults communicate across Twitter and Facebook. By building age prediction models specifically for youth and young adult age groups that are at risk for negative health behaviors, our results can help inform better targeting of public health surveillance and education efforts online.

## Supporting information

S1 Table Description of metadata and linguistic features. (DOCX)

S2 Table Tests of different classifiers. A) Based on Accuracy B) Based on F-1 Score.
(DOCX)
