# Supplementary material for: Predicting age groups of Twitter users based on language and metadata features
Source: PLoS One. 2017 Aug 29;12(8):e0183537. doi: 10.1371/journal.pone.0183537 (PMC5574558; doi:10.1371/journal.pone.0183537)
Supplement: S1 Table — (DOCX) [file pone.0183537.s001.docx]

S1 Table. Description of Metadata and Linguistic Features

| **Variable Name** | **Description** | **Twitter Default Variable** | **Distributional Features^a^** | **Normalized Tweet Features^b^** |
| --- | --- | --- | --- | --- |
| ***Metadata Features*** | | | | |
| default_profile | When true, indicates that the user has not altered the theme or background of their user profile. | 1 | 0 | 0 |
| default_profile_image | When true, indicates that the user has not uploaded their own avatar and a default egg avatar is used instead. | 1 | 0 | 0 |
| followers_count | The number of followers this account currently has. Under certain conditions of duress, this field will temporarily indicate “0.” | 1 | 0 | 0 |
| friends_count | The number of users this account is following (AKA their “followings”). Under certain conditions of duress, this field will temporarily indicate “0.” | 1 | 0 | 0 |
| statuses_count | The number of tweets (including retweets) issued by the user. | 1 | 0 | 0 |
| verified | When true, indicates that the user has a verified account. | 1 | 0 | 0 |
| favourites_count | The number of tweets this user has favorited in the account’s lifetime. British spelling used in the field name for historical reasons | 1 | 0 | 0 |
| listed_count | The number of public lists that this user is a member of. | 1 | 0 | 0 |
| geo_enabled | When true, indicates that the user has enabled the possibility of geotagging their Tweets. | 1 | 0 | 0 |
| favorite_count | Indicates approximately how many times this Tweet has been liked by Twitter users. | 1 | 1 | 1 |
| hashtag_count | Number of hashtags posted in tweet | 1 | 1 | 1 |
| media_count | Number of media objects posted in tweet | 1 | 1 | 1 |
| mention_count | Number of user mentions posted in tweet | 1 | 1 | 1 |
| retweet_count | Number of times this Tweet has been retweeted. | 1 | 1 | 1 |
| url_count | Number of URLs posted in tweet | 1 | 1 | 1 |
| tweet_velocity | The average number of tweets since the account was created. | 0 | 0 | 0 |
| days_created | The number of days the creation of account. | 0 | 0 | 0 |
| desc_len | The number of terms in the Twitter account "description" field | 0 | 0 | 0 |
| follow_per_friend | The number of an account's followers divided by the number of an account's friends | 0 | 0 | 0 |
| coord_flag | Indicates if a tweet has coordinates representing the geographic location | 0 | 1 | 1 |
| place_flag | When present, indicates that the tweet is associated (but not necessarily originating from) a Place (Twitter object) | 0 | 1 | 1 |
| ***Linguistic Derived Features*** | | | | |
| num_cap | Number of terms in tweet that are in all caps | 0 | 1 | 1 |
| num_emoji | Number of emoji present in tweet | 0 | 1 | 1 |
| num_exclaim | Number of exclamation points in a tweet | 0 | 1 | 1 |
| num_laugh | Number of "laughing" terms used (e.g. 'lol', 'lmoa', etc.) in tweet | 0 | 1 | 1 |
| num_question | Number of question marks present in a tweet | 0 | 1 | 1 |
| num_reap | Number of terms with alphabetical lengthening (e.g. "sickkkkk") | 0 | 1 | 1 |
| num_swear | Number of swear words in a tweet. Used a non-exhaustive list of over 300+ english swear words/terms from "2g1c" to "zoophilia". | 0 | 1 | 1 |
| num_tot_punc | Number of words in tweet. | 0 | 1 | 1 |
| num_wrds | Number of top 100 most negatively correlated terms on Facebook with the 30+ age category (Schwartz et al. 2013) in a tweet. | 0 | 1 | 1 |
| num_wwbp_adult_negative | Number of top 100 most positively correlated terms on Facebook with the 30+ age category (Schwartz et al. 2013) in a tweet. | 0 | 1 | 1 |
| num_wwbp_adult_positive | Number of top 100 most negatively correlated terms on Facebook with the 23-29 age category (Schwartz et al. 2013) in a tweet. | 0 | 1 | 1 |
| num_wwbp_mid_negative |  | 0 | 1 | 1 |
| num_wwbp_mid_positive | Number of top 100 most positively correlated terms on Facebook with the 23-29 age category (Schwartz et al. 2013) in a tweet. | 0 | 1 | 1 |
| num_wwbp_ya_negative | Number of top 100 most negatively correlated terms on Facebook with the 19-22 age category (Schwartz et al. 2013) in a tweet. | 0 | 1 | 1 |
| num_wwbp_ya_positive | Number of top 100 most positively correlated terms on Facebook with the 19-22 age category (Schwartz et al. 2013) in a tweet. | 0 | 1 | 1 |
| num_wwbp_youth_negative | Number of top 100 most negatively correlated terms on Facebook with the 13-18 age category (Schwartz et al. 2013) in a tweet. | 0 | 1 | 1 |
| num_wwbp_youth_positive | Number of top 100 most positively correlated terms on Facebook with the 13-18 age category (Schwartz et al. 2013) in a tweet. | 0 | 1 | 1 |
| wrd_gt6 | Number of words greater than 6 characters in tweet. | 0 | 1 | 1 |
| wrd_stan | Number of standard English words in tweet. Uses wordlist form the Moses Machine Translation toolkit (Koehn et al. 2007) through the Python NLTK library. | 0 | 1 | 1 |
| Term frequencies | Frequency counts for unique tokens and bi-grams across all tweets (stopword removed). Resulted in 12,038 unique tokens (from "!!!" to "zoom"). | 0 | 0 | 0 |
| Stemmed term frequencies | Frequency counts for unique stemmed terms across all tweets. Resulted in 6,149 unique terms. | 0 | 0 | 0 |
| Synset term frequencies | WordNet synsets for all unique standard words in the tweet corpus were extracted. For each corresponding synset, the features here represent the frequency count of synonym words found within each tweet. All synsets for a word were considered regardless of its use in the tweet, resulting in 19,965 unique features. | 0 | 0 | 0 |

^a^ To capture the distributional characteristics of a user's tweet usage, we also calculated features of summary statistics on these tweet-level counts across all a user's collected tweets (mean, median, minimum, maximum, median absolute deviation, skew, variance).

^b^ These features also had versions created that normalize counts for tweet length (bounded by 0 to 1).
