# Supplementary material for: Predicting age groups of Twitter users based on language and metadata features
Source: PLoS One. 2017 Aug 29;12(8):e0183537. doi: 10.1371/journal.pone.0183537 (PMC5574558; doi:10.1371/journal.pone.0183537)
Supplement: S2 Table — A) Based on Accuracy B) Based on F-1 Score. (DOCX) [file pone.0183537.s002.docx]

S2 Table. Tests of Different Classifiers

1. ***Based on Accuracy***

| **Model** | **Test Set Accuracy** | **Test Set Rank** | **Avg. CV Accuracy** | **CV Rank** |
| --- | --- | --- | --- | --- |
| Logistic Regression | 73.54% | 1 | 74.52% | 2 |
| Logistic Elastic Net | 71.65% | 2 | 73.49% | 1 |
| SVM | 70.87% | 3 | 73.02% | 3 |
| Random Forests | 70.39% | 4 | 68.29% | 4 |
| AdaBoost | 68.03% | 5 | 68.18% | 5 |
| Extra Trees | 67.40% | 6 | 64.99% | 6 |
| Dummy | 51.50% | 7 | 51.44% | 7 |

1. ***Based on F1-score***

| **Model** | **Test Set F1** | **Test Set Rank** | **Avg. CV F1** | **CV Rank** |
| --- | --- | --- | --- | --- |
| Logistic Regression | 73.85% | 1 | 74.52% | 2 |
| Logistic Elastic Net | 71.65% | 2 | 73.43% | 1 |
| SVM | 71.15% | 3 | 72.76% | 3 |
| Random Forests | 68.24% | 4 | 67.74% | 5 |
| AdaBoost | 67.98% | 5 | 65.19% | 4 |
| Extra Trees | 64.80% | 6 | 61.23% | 6 |
| Dummy | 37.95% | 7 | 39.43% | 7 |
